# A Step‐by‐Step Guide to Successful Multiparametric Microbiota Flow Cytometry

**DOI:** 10.1002/cpz1.70460

**Published:** 2026-09-04

**Authors:** Toni Sempert, Lisa Budzinski, Robin Kempkens, Gi‐Ung Kang, René Maier, Hyun‐Dong Chang

**Affiliations:** ^1^ German Rheumatology Research Center Berlin – A Leibniz Institute Berlin Germany; ^2^ Department for Cytometry, Institute of Biotechnology Technische Universität Berlin Berlin Germany

**Keywords:** host–microbiota interactions, microbiome, microbiota phenotyping, multiparametric flow cytometry

## Abstract

The role of the human microbiota in host health is an important area of research. Conventional characterization of the complex microbial communities colonizing the human body is primarily performed using culture‐based or high‐throughput sequencing approaches, which have improved general understanding yet lack cellular features relevant for interaction and function at the single‐cell level. Flow cytometry is an established tool for single‐cell analysis, and recent improvements, such as increased resolution of small cells and particles, offer possibilities for characterizing complex microbial communities. Here, we describe an updated protocol to characterize complex microbial communities derived from human stool samples at the single‐cell level by multiparametric microbiota flow cytometry. Our protocol covers the process from the isolation of bacteria from stool, preparation of cryo‐stocks, staining procedure for phenotypic features of the bacteria, and flow cytometric analysis. Our phenotypic characterization includes light scattering properties, quantitative DNA staining, isotype‐specific staining of host–antibody coating, and profiling of bacterial cell surface sugar moieties. In addition, we highlight the importance of determining the bacterial load for improved biological interpretation. External staining controls ensure reproducibility and quality controls. Additionally, we suggest a downstream analysis pipeline involving segmentation of multivariate data by a self‐organized map (SOM) and machine learning to generate specific microbiota fingerprints. Optionally, bacteria with specific phenotypic characteristics can be isolated by fluorescence‐activated cell sorting (FACS) for further investigation. Our protocol can be applied to any microbial community or sample source and offers the flexibility to be expanded with additional phenotypic markers. © 2026 The Author(s). *Current Protocols* published by Wiley Periodicals LLC.

**Basic Protocol 1**: Preparation of cryo‐preserved bacterial stocks from a single‐cell microbiota suspension derived from native human stool sample

**Basic Protocol 2**: Staining protocol for cryo‐preserved microbiota stocks and acquisition using a flow cytometer

## INTRODUCTION

The human microbiota is a complex community colonizing every surface of the human body exposed to the environment and plays an important role in human health and disease. To date, characterization of the microbiota is primarily based on high‐throughput sequencing such as 16S rRNA gene sequencing or multiomics approaches. These analyses established that changes in the microbiota are associated with various diseases (Hou et al., 2022). However, the role the microbiome plays in human disease still remains unclear. Therefore, resolving how the microbiota contributes to disease requires an understanding of the function of the microbiota and its individual members (Budzinski, Von Goetze, et al., 2024).

Single‐cell phenotyping by flow cytometry is a well‐established and widely used tool to study individual cells in a high‐throughput manner. Flow cytometry has been successfully applied to characterize microbial community structure and dynamics from environmental, mouse, and human samples at the single‐cell level (Koch et al., 2013; Props et al., 2016; Rubbens et al., 2021a; Schmiester et al., 2022; Vandeputte et al., 2017; Zimmermann et al., 2016). However, the analyses were restricted to light scattering characteristics and DNA staining and lacked interrogation of parameters providing information on bacterial crosstalk with the host immune system or functionality of the microbial community. Multi‐parameter microbiota flow cytometry addresses these gaps to deliver a more comprehensive phenotypic characterization of the human microbiome (Budzinski et al., 2025, 2026; Budzinski, Radbruch, et al., 2024; Budzinski, Sempert, et al., 2024; Budzinski, Von Goetze, et al., 2024).

Here, we present an updated workflow for single‐cell bacterial phenotyping adapted from previously established protocols that includes the following:

•isolation protocol to prepare a microbial single‐cell suspension and cryo‐preserved stocks for multi‐parametric flow cytometry (Budzinski, Radbruch, et al., 2024)
•staining for host‐derived immunoglobulins coating the bacterial surface to investigate the host–microbiota crosstalk (Budzinski, Radbruch, et al., 2024)
•staining of the bacterial surface for specific sugar moieties with plant‐derived lectins to interrogate adaptations of bacteria to the microenvironment,
•absolute quantification of bacteria to determine the microbial load (Vandeputte et al., 2017)
•an optimized data analysis pipeline for obtained multivariate data (Budzinski et al., 2025)

The obtained multiparametric data, including morphological and phenotypic information of microbial cells, can be analyzed by different algorithms to generate microbiota fingerprints. These microbiota fingerprints can be further used to investigate microbial communities in environmental contexts such as aqua farms (Heyse et al., 2021) and wastewater (Görnt et al., 2025; Günther et al., 2012) or as a tool in human health to describe disease‐specific patterns or monitor and predict therapy responsiveness (Budzinski et al., 2025, 2026; Budzinski, Sempert, et al., 2024; Rubbens et al., 2021a).

## CAUTIONS

Untested human stool samples can contain spore‐forming and potentially pathogenic bacteria. We recommend sample preparation under a sterile bench and application of filter tips to avoid (cross‐) contamination.

## STRATEGIC PLANNING

**Figure 1:**
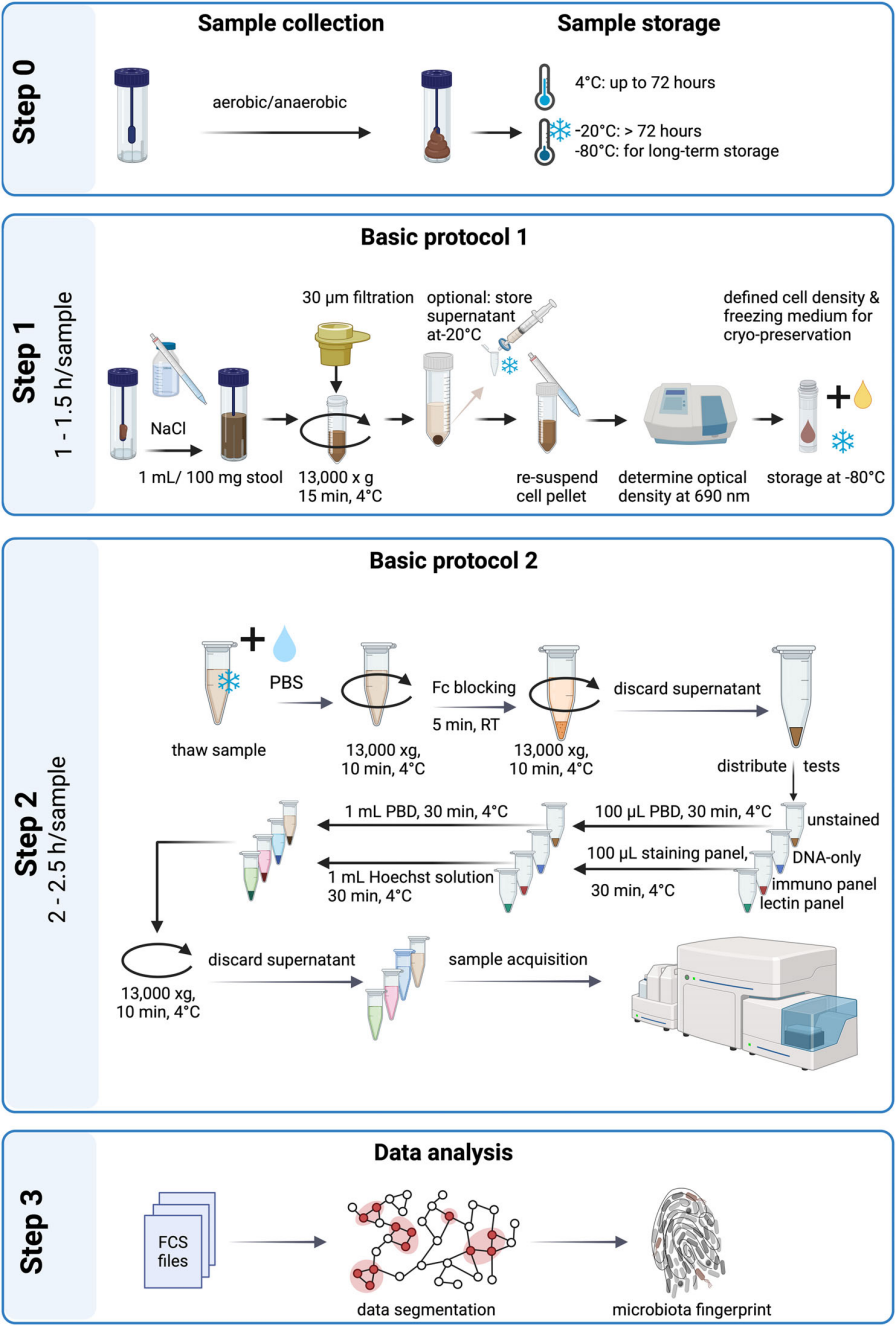
Schematic diagram of modular multi‐parametric microbiota flow cytometry workflow. (Created using BioRender. Sempert, T. (2026); https://BioRender.com/d3vhl89)

Here, we present an updated comprehensive and modular workflow for multi‐parametric microbiota flow cytometry (Fig. 1), extending previous protocols (Budzinski, Radbruch, et al., 2024), which can be performed on different days. To ensure reliable and reproducible results, some key considerations should be made to avoid batch effects before starting.

### Sample Collection Logistics

All native stool samples must be collected and stored under the same conditions. Samples can be stored at 4°C for up to 72 h before processing or for long‐term storage at −20°C or ideally at −80°C (Budzinski, Radbruch, et al., 2024; Krause et al., 2024).

### Pre‐experiment Optimization

Prior to screening, it is crucial to titrate all detection antibodies, lectins, and DNA dyes during the panel development to determine the optimal reagent concentration and achieve the best signal‐to‐noise ratio (Cossarizza et al., 2017). In addition, it is recommended to perform a small titration when starting with new batches of reagents to ensure optimal titers. Optimal titers vary depending on the sample type (e.g., stool sample vs. saliva) and have to be determined specifically for each sample type. Moreover, to ensure specific detection of bacterial cell surface sugar moieties by plant lectins, blocking experiments should be performed by incubating the lectin with the target sugar before staining (see suppl. Fig. 1). The same applies to the specificity of the detection antibodies for anti‐human immunoglobulin staining. Instrument settings and performance should be standardized to ensure data quality during sample acquisition.

### External Control (Anchor Sample)

One or more human stool samples with a known and distinct staining pattern or bacterial mock communities with less complexity (Cichocki et al., 2020) should be included in all cytometric measurements as an external control to control the staining procedure and optionally for batch effect correction between multiple runs during data analysis. The amount of this external control must be sufficient to conduct the entire experiment or study.

### Reagents

A sufficient quantity of the respective antibody clone or lectin should be available to avoid using different clones during data acquisition.

*NOTE*: Stool samples were provided by all donors under approval of the local ethics committee of the Charité Berlin (approval reference: EA4/014/20; EA4/247/20) and in accordance to the Helsinki II Declaration.

*NOTE*: All protocols involving animals must be reviewed and approved by the appropriate Animal Care and Use Committee and must follow regulations for the care and use of laboratory animals. Appropriate informed consent is necessary for obtaining and using human study material.

## Basic Protocol 1 PREPARATION OF CRYO‐PRESERVED BACTERIAL STOCKS FROM A SINGLE‐CELL MICROBIOTA SUSPENSION DERIVED FROM NATIVE HUMAN STOOL SAMPLE

This protocol aims to prepare a microbial single‐cell suspension from a native human stool sample. In short, samples are resuspended in a defined volume of 0.9% NaCl (1 mL buffer/100 mg stool), filtered through 30 µm to remove food‐derived fibers followed by preparation of cryo‐preserved bacterial stocks with a defined optical density as the final protocol output.

### Materials

Human stool sample (minimum 0.5 g)
0.9% (w/v) NaCl, 0.22‐µm sterile‐filtered and autoclaved (see recipe)
Freezing medium, 0.9% (w/v) NaCl solution with 25% (v/v) glycerol (see recipe)

Laminar flow hood
Precision balance (Kern, cat. no. PCB 350‐3)
Sterile spatula
Filter, 30 µm (MACS SmartStrainer, Miltenyi Biotec, cat. no. 130‐098‐458 or Celltrics filter, Sysmex, cat. no. 04‐0042‐2316)
Falcon tube, 50 mL
Sorvall ultracentrifuge RC‐6 plus (Thermo Fisher Scientific, cat. no. 46915)
Syringe filter, 0.22 µm
Flat‐bottom plate, 96‐well
Microplate photometer, 96‐well (Multiskan FC, Thermo Fisher Scientific, cat. no. 51119000)
Safe‐seal tube, 2 mL (Eppendorf)
Multichannel pipette, 200 µL
Pipettes,10, 20, 200, and 1000 µL
Pipette tips with filter, 10, 20, 200, and 1250 µL
Benchtop centrifuge (Fresco 17, Thermo Fisher Scientific, cat. no. 75002420)
Biobank tubes (LX1000, LVL technologies, cat. no. 2DSC‐X10‐BR‐NS‐SLC‐S)

1 Before starting the procedure:
  a. Native stool samples should be stored for a maximum of 72 h at 4°C after sample collection or at −20°C/ −80°C, in case the sample storage exceeds 72 h.
  b. Precool the ultracentrifuge to 4°C.
  c. Split the original sample in case other analyses are planned, such as metabolomics or metaproteomics, which also require native stool.
2 Determine the weight of the (partitioned) native stool sample by subtracting the weight of the empty pre‐weighed sample tube from the weight of the sample‐containing tube.
  We recommend proceeding with the protocol under a laminar flow hood or, if necessary, using an anaerobic chamber to maintain anaerobic conditions.
3 Add 0.22‐µm sterile‐filtered and autoclaved 0.9% (w/v) NaCl to the sample‐containing tube, at 1 mL/100 mg stool.
4 Dissolve the sample by vigorous vortexing or mechanical disruption using a sterile spatula in case vortexing is not sufficient.
5 Filter the bacterial suspension through a 30‐µm filter to remove food residues into a suitable tube for subsequent centrifugation.
  We usually use Miltenyi MACS SmartStrainer with a maximum reservoir volume of 16 mL.
  For bacterial suspensions of smaller volumes, we recommend a 30‐µm filter from Sysmex.
6 Spin down the filtered bacterial suspension for 15 min at 13,000 × g and 4°C using the pre‐cooled centrifuge and discard the fecal supernatant.
  Caution: It is critical to use tubes that withstand high centrifugation forces and do not break.
  Optional: Filter the fecal supernatant through a 0.2‐µm filter into a new tube if the supernatant should be used for further analysis, e.g., to analyze fecal antibodies or other analytes, and store it under the required conditions (−20°C or −80°C).
  Caution: Please be careful while filtering, as the syringe filter can clog and excess pressure may cause fecal supernatant spillage.
7 Resuspend the cell pellet in the same volume as that added in step 3 and as described in step 4.
8 Determine the optical density (OD) of the bacterial suspension at 690 nm.
  a. Use the 0.9% (w/v) NaCl as the blank reference.
  b. Suggested dilution for bacterial suspension: 1:20 and 1:50
    We recommend using a 96‐well flat‐bottom plate when multiple stool samples are processed at the same time and shaking the plate at medium velocity before OD measurements.
9 Calculate the volume required for the defined OD_690 nm_ values of 0.4/mL and 2.0/mL using the following formula:
$$x\;\mu L\;\mathrm{for}\;\mathrm{OD}\frac{0.4}{mL}=\;\frac{0.4\times 1000}{{OD}_{690\;nm}}$$
$$x\;\mu L\;\mathrm{for}\;\mathrm{OD}\frac{2.0}{mL}=\;\frac{0.4\times 1000}{{OD}_{690\;nm}}$$
  We use defined ODs for our experiments (OD_690 nm_ of 0.4/mL for flow cytometric measurements and OD_690 nm_ of 2.0/mL as a backup for long‐term storage) to ensure similar cell densities across donors and reproducibility.
10 For flow cytometric measurement stocks, transfer the determined volume, usually between 25 and 75 µL, of the microbiota suspension with OD_690 nm_ of 0.4 into a new 2‐mL safe‐seal tube or an equivalent cryo‐tube; add 1 mL freezing medium, invert for mixing, and immediately store the cryo‐preserved samples at −80°C for further analysis and continue with Basic protocol 2.
  If the required volume exceeds 300 µL, we recommend an additional centrifugation step to reduce the volume of the bacterial suspension and then resuspend the cell pellet in 1 mL of freezing medium. This ensures better cryo‐preservation and prevents the freezing medium from being significantly diluted by NaCl solution.
  Optional: Proceed with Basic protocol 2 if bacteria should be stained in their freshly isolated state without cryo‐preservation.
11 For long‐term storage of OD_690 nm_ 2.0 samples, transfer the sample resuspended in the determined volume into a new 2‐mL safe‐seal tube and centrifuge for 10 min at 13,000 × g and 4°C.
12 Discard the supernatant, resuspend the cell pellet in 500 µL freezing medium, transfer the cell suspension into a cryo‐tube for long‐term storage, and immediately store at −80°C.
  The recommended cryovials have a maximum volume of 1 mL, which is why we recommend resuspending the cell pellet in 500 µL freezing medium and adding 500 µL of buffer during the thawing process to dilute the freezing medium (the glycerol is harmful to bacteria at higher concentrations).

## Basic Protocol 2 STAINING PROTOCOL FOR CRYO‐PRESERVED MICROBIOTA STOCKS AND ACQUISITION USING A FLOW CYTOMETER

The aim of this protocol is to perform surface staining of previously cryo‐preserved microbiota stocks (optionally fresh sample) and acquisition of stained samples using a flow cytometer. After a blocking step, to avoid nonspecific binding of detection antibodies, staining with isotype‐specific monoclonal anti‐human antibodies is conducted to detect bacteria coated with host immunoglobulins. Staining with a plant lectin panel is performed to profile bacterial cell surface sugar moieties. Additionally, quantitative DNA staining is performed to differentiate bacteria from debris with a viable cell dye (Hoechst33342) and determine DNA content. Counting beads are added, or volumetric measurement is performed to determine the microbial load. After sample acquisition, the multivariate data obtained can be analyzed by various algorithms to describe the microbiota phenotype further.

### Materials

Cryo‐preserved bacterial stocks from Basic Protocol 1
PBS (Th. Geyer GmbH & Co. KG, cat. no. 8462‐5PCE)
F_c_‐blocking solution (see recipe)
PBS/BSA [0.2% (w/v) BSA; PAN Biotech GmbH, cat. no. P06‐1391500]
PBD (DNase I‐containing PBS/BSA; see recipe)
Immunoglobulin staining mix (see recipe)
Lectin staining mix (see recipe)
Hoechst33342 solution (see recipe)
Megamix‐Plus FSC beads (BioCytex, cat. no. 7803)
Sphero Rainbow Particles (Waters Biosciences, cat. no. 559123)
FluoSpheres Carboxylate‐Modified Microspheres – 1 µm, blue (Invitrogen, cat. no. 10358482)
Precision Counting Beads (BioLegend, cat. no. 424902)

Benchtop centrifuge (Fresco 17, Thermo Fisher Scientific, cat. no. 75002420)
Safe‐seal tube, 2 mL (Eppendorf)
Pipettes, 10, 20, 200, and 1000 µL
Pipette tips with filter, 10, 20, 200, and 1250 µL
Multilaser flow cytometer [UV laser (355 nm), violet laser (405 nm), blue laser (488 nm), yellow laser (561 nm), and red laser (637 nm)]

#### Microbiota staining protocol

1 Before starting the procedure:
  a. All buffers must be filtered through a 0.22‐µm filter.
  b. Precool the centrifuge to 4°C.
  c. Sample staining is performed in 2‐mL safe‐seal tubes. If staining is conducted in 96‐well plates with lower volume, please adjust the volume of the staining mix accordingly.
  d. The protocol applies to microbiota samples of OD_690nm_ 0.4, corresponding to approximately 10 tests.
  e. For a more efficient workflow, we recommend preparing the blocking solution and staining cocktails of the immunoglobulin and lectin panels during step 2 (see recipe).
  f. We highly recommend including a known sample as a technical anchor control. This sample can be used to control the staining procedure and for batch effect correction (see Statistical Analysis and Understanding the Results; Suppl. Fig. 2).

**Figure 2:**
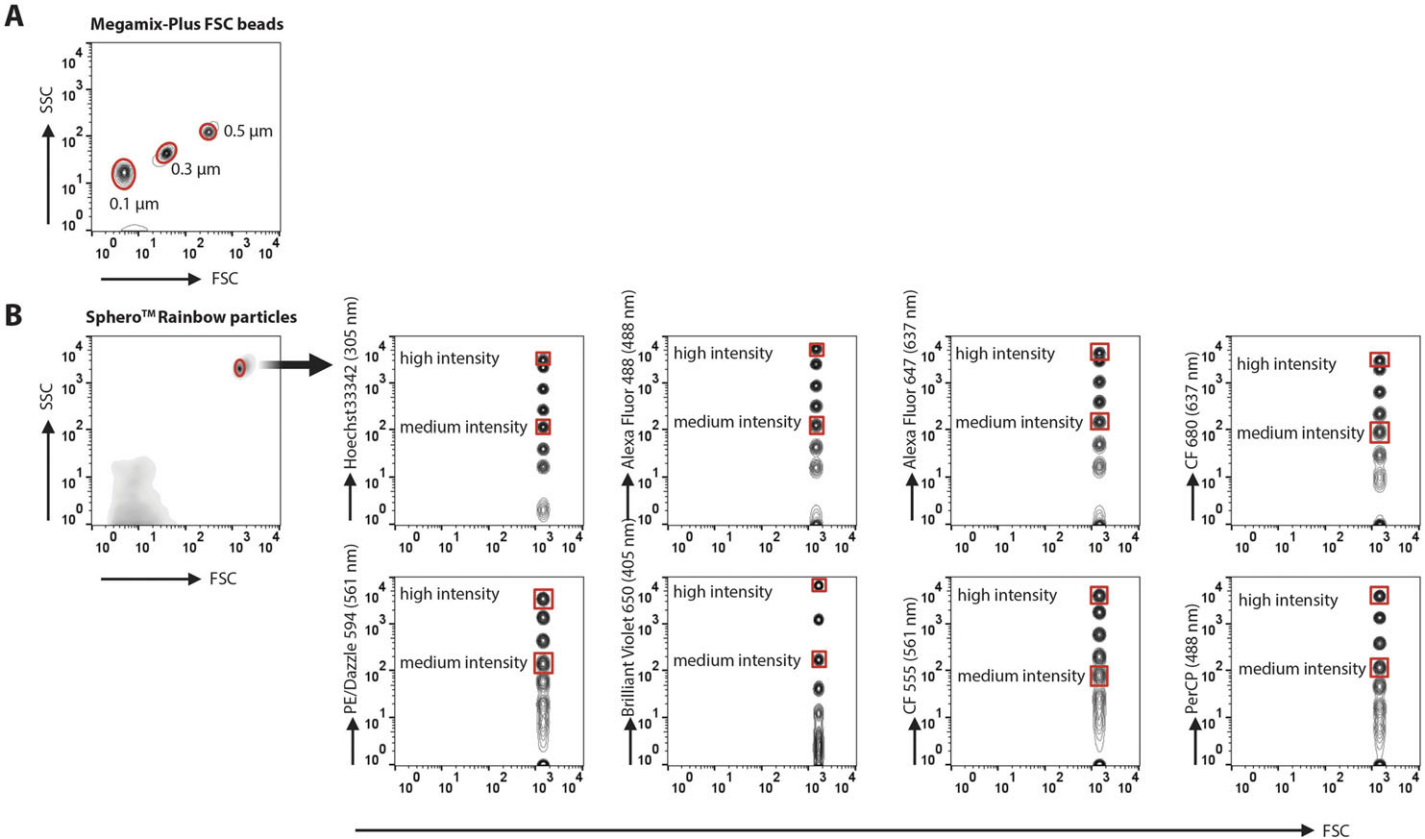
Cytometer setup for instruments not performing an automatic calibration. **(A)** Megamix‐Plus FSC beads and **(B)** Sphero Rainbow particles are applied to ensure comparability across measurements. The lasers have to be adjusted until the respective beads are located in their predefined gate for scatter and each fluorescence channel.

2 Add 1 mL PBS to the frozen stock while thawing at room temperature. Immediately after thawing, spin down the cells for 10 min at 4°C, 13,000 × g.
  Caution: Glycerol is harmful to bacteria and must be removed immediately after thawing.
  When processing a larger number of bacterial stocks (n > 10) simultaneously, we recommend thawing the cells on ice (∼5–10 min) to reduce toxicity effects, as the freezing medium contains glycerol.
3 Discard the supernatant, resuspend the cell pellet in 500 µL blocking solution, and incubate for 5 min at room temperature. Add 1 mL PBS/BSA and spin down for 10 min at 4°C, 13,000 × g.
4 Prepare prelabeled 2‐mL safe‐seal tubes preloaded with the respective staining cocktail, immunoglobulin, and lectin panel in 95 µL PBD (DNase I‐containing PBS/BSA) and 95 µL PBD only for each test and staining control, respectively. Staining controls comprise an unstained sample and a DNA‐only stained sample.
5 Discard the supernatant of the pelleted samples, and resuspend the cell pellet of each sample in 50 µL PBD. To each prepared tube (step 4, unstained, DNA‐only stained, immunoglobulin staining, and lectin staining), add 5 µL bacterial suspension, corresponding to a final OD_690nm_ of 0.04, and incubate for 30 min at 4°C in the dark.
6 Add 1 mL Hoechst 33342 solution to the DNA‐only stained samples and the respective panel‐stained samples. Add 1 mL PBD to the unstained sample. Incubate all samples for 30 min at 4°C in the dark.
7 Top up with 900 µL PBS/BSA and spin down for 10 min at 4°C, 13,000 × g.
8 Remove the supernatant carefully and resuspend the cell pellet in 1 mL PBS/BSA.

#### Instrument setup and microbiota flow cytometry

9 Before sample acquisition, align the flow cytometer to ensure consistent and reproducible instrument performance. For cytometers not performing an automatic calibration to ensure day‐to‐day reproducibility, we highly recommend performing steps 9a and 9b:
  a. Apply Megamix‐Plus FSC beads to adjust the laser used for scattering (Fig. 2A).
    We recommend using predefined gates to ensure instrument performance and comparability across experiments or multiple measurement batches. The gates should be set once, and the instrument then adjusted for each measurement in such a way that the beads are located within the predefined gates.
  b. Adjust the lasers used for fluorescence by adding Sphero Rainbow Calibration Particles (Fig. 2B).
    We recommend using predefined gates for high and intermediate signal intensity as well as the application of the anchor control to ensure comparable instrument performance. The gates are set once for each used fluorescence channel with specific signal intensities for the high‐ and medium‐intensity gates (these values are instrument‐specific). For subsequent measurements, the instrument should be adjusted so that the beads are located within the predefined gates.
10 Use the unstained sample to determine the average event rate and dilute the cells sufficiently to achieve an event rate not higher than 10,000 events/s during acquisition. Apply this dilution to the DNA‐only stained sample and panel samples.
11 For sample acquisition, record 300,000 DNA‐positive events for each sample. Use the unstained control to determine DNA positivity.
  a. We suggest the following order during acquisition: unstained sample, DNA‐only sample, and panel sample.
  b. Depending on the flow cytometer, we recommend performing a backflush and running water between samples or performing rinse cycles to prevent sample carryover.
  c. Perform bead controls regularly using calibration beads to ensure stable cytometer performance during sample acquisition, at least before and after acquisition of all samples.
  Optional: Spike in size beads (e.g., 1‐µm bright blue beads) into your sample to control scatter properties during acquisition (see Suppl. Fig. 3).

**Figure 3:**
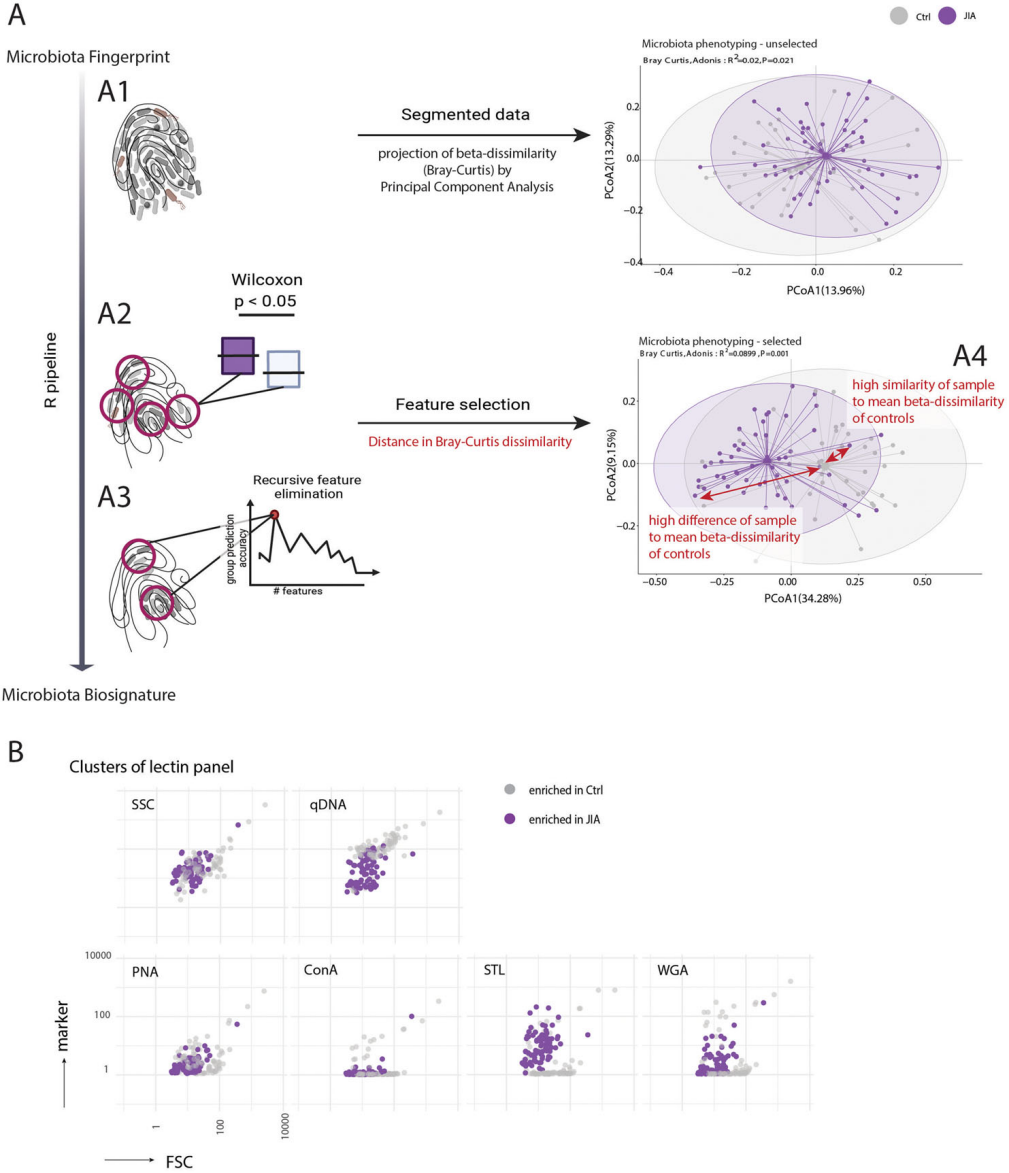
Exemplary analysis workflow for multivariate microbiota phenotyping data. **(A)** R analysis pipeline to select cohort‐ or group‐specific features from the microbiota fingerprint (A1) to obtain a microbiota biosignature (A2–A4). Clusters were filtered by (A2) Wilcoxon rank‐sum test and (B3) recursive feature elimination to select the significant and most robust clusters defining the specific microbiota phenotype signature represented for all samples by their β‐diversity (Bray–Curtis dissimilarity) projected by a principal component analysis (PCoA) (A4). **(B)** Representation of selected phenotypic clusters for their location in 2D dot plot of all lectin markers included in the panels (y‐axis) against forward scatter (FSC, x‐axis). The same visualization can be applied for anti‐human immunoglobulins. All clusters enriched in juvenile idiopathic arthritis (JIA) are depicted in purple; clusters enriched in controls are depicted in grey (Budzinski, Sempert, et al., 2024).

**Figure 4:**
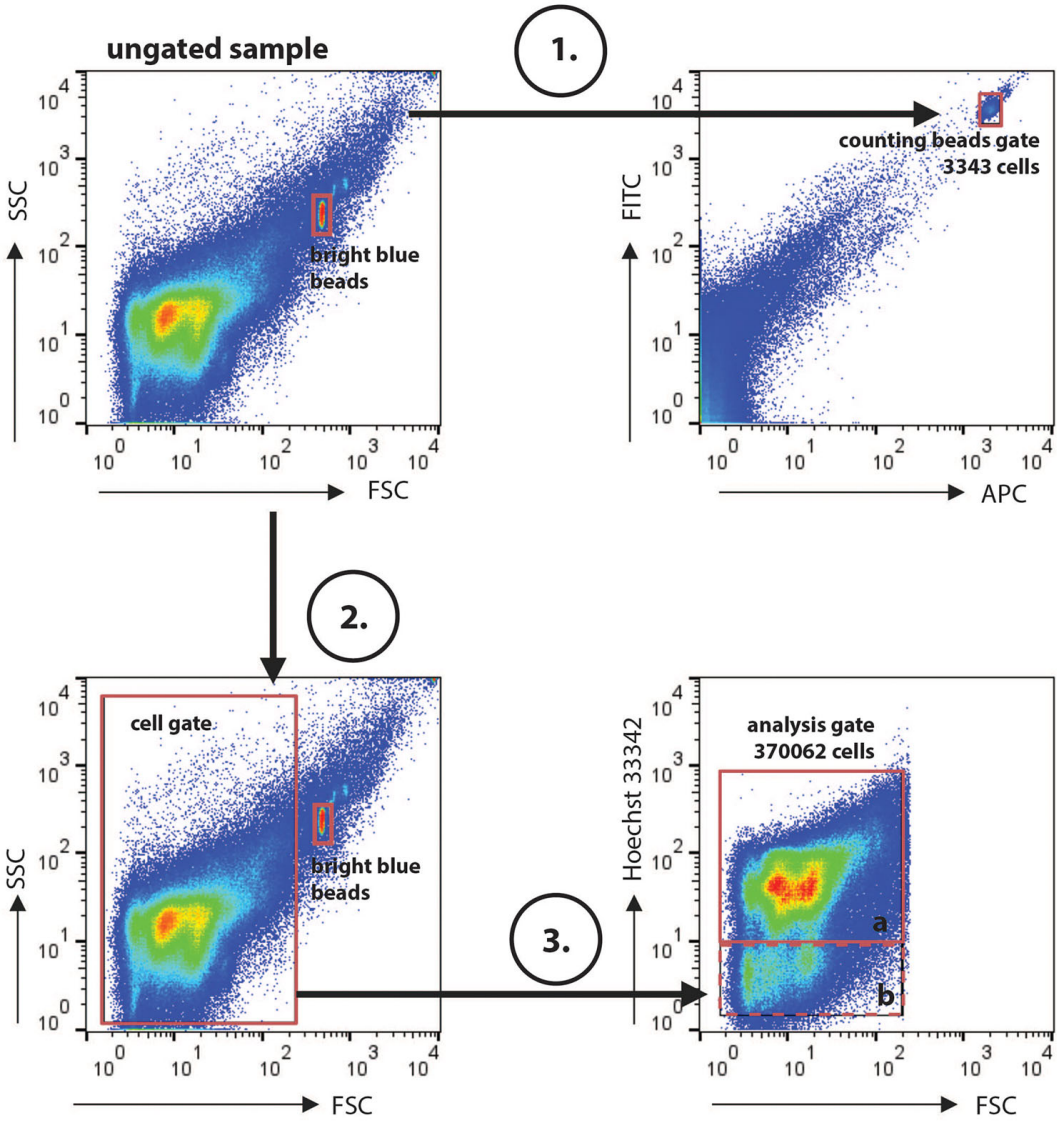
Gating strategy for absolute quantification of microbial load using counting beads. ① The ungated sample is displayed in two fluorescence channels (FITC on the y‐axis, APC on the x‐axis) to define the counting bead gate and extract the bead count. ② A cell gate is set to exclude electronic noise and cell debris. Events falling below this fluorescence threshold are excluded. ③ The final analysis gate is applied to the remaining events [defined by forward scatter (x‐axis) vs. marker of interest (y‐axis; here Hoechst 33342 dye on DNA^high^ events or all events except noise)], and the cell count within this gate is extracted. The total bacterial count is calculated by multiplying the cell count by the ratio of the bead count to the known bead concentration, as well as the sample volume. Bright blue beads with a defined size were included to control scatter properties during acquisition. All gates were defined and applied using FlowJo software.

12 Perform cell counting.
  a. If the cytometer does not have a volumetric cell counting function, we recommend adding a defined volume of counting beads to each sample for cell count determination later (see Statistical analysis and Understanding the Results; Fig. 4).
    We recommend 20 µL counting beads for a 200 µL sample.
  b. If the cytometer possesses a volumetric cell counting function, export the acquired volume/cell concentration to quantify the bacterial load.

## REAGENTS AND SOLUTIONS

### DNase I aliquots

100 mg DNase I (10 mg/mL final; Roche, cat. no. 11284932001; stable at 4°C)
10 mL ddH_2_O
Mix carefully to avoid foaming.
Aliquot 125 µL suspension into 0.2‐ or 0.5‐mL safe‐seal tubes and store at 4°C until further use.

### Fc‐blocking solution

This solution should contain 5× to 10× your detection antibodies and respective isotypes.

Staining volume per cryo‐preserved stock: 500 µL
490 µL PBS
10 µL purified mouse IgG_1_ (20 µg/mL final; clone: IS5‐21F5, Miltenyi Biotech, cat. no. 130–106–545)
Store the staining mixture on ice until use.

### Freezing medium

4.5 g NaCl [0.9% (w/v) final; Roth, cat. no. 3957.2]
375 mL ddH_2_O
Mix well until NaCl is dissolved.
125 mL glycerol [25% (v/v) final; Roth, cat. no. 3783.1]

We recommend adding glycerol to the salt solution by weighing it out (157.5 g) due to its high viscosity.

Autoclave the freezing medium and store at 4°C.

### Hoechst33342 solution

12.5 µL Hoechst 33342 (5 µM final; Thermo Fisher Scientific, cat. no. 62249)
50 mL PBD

### Immunoglobulin staining mix

Before you start, vortex all antibodies vigorously and spin down for 1 min at 17,000 × g to avoid the transfer of fluorochrome aggregates to the staining mix.

Staining volume per test: 100 µL
96 µL PBD
anti‐human detection antibodies 1:100 (v/v)
anti‐human IgA_1_‐Alexa Fluor 647 (clone: B3506B4, Southern Biotech, cat. no. 9130–31)
anti‐human IgA_2_‐Alexa Fluor 488 (clone: A9604D2, Southern Biotech, cat. no. 9140–30)
anti‐human IgG‐PE/Dazzle 594 (clone: HP6017, BioLegend, cat. no. 409324)
anti‐human IgM‐Brilliant Violet 650 (clone: MHM‐88, BioLegend, cat. no. 314526)
Store the staining mixture on ice, protected from light, until distribution.

### Lectin staining mix

Before you start, vortex all fluorochrome‐conjugated reagents vigorously and spin down for 1 min at 17,000 × g to avoid transferring fluorochrome aggregates to the staining mix.

Staining volume per test: 100 µL
96.25 µL PBD
2 µL anti‐Biotin PerCP [1:50 (v/v) final; clone: Bio3‐18E7, Miltenyi Biotech, cat. no. 130–133–293]
0.5 µg biotinylated Solanum Tuberosum Agglutinin (STL) (0.005 µg/µL final; Vector Laboratories/Biozol, cat. no. B‐1165)
Pre‐incubate the biotinylated STL and anti‐biotin PerCP antibody briefly with 20 µL PBD for 5 min and add the remaining 76.25 µL PBD.
0.5 µg Peanut Agglutinin‐CF 488 (PNA) (0.005 µg/µL final; Biotium, cat. no. 29060)
0.5 µg Concanavalin A‐CF 680 (Con A) (0.005 µg/µL final; Biotium, cat. no. 29020–1),
0.25 µg Wheat Germ Agglutinin‐CF 555 (WGA) (0.0025 µg/µL final; Biotium, cat. no. 29076–1)
Store the staining mixture on ice, protected from light, until distribution.

### NaCl solution, 0.9% (w/v)

4.5 g NaCl [0.9% (w/v) final; Roth, cat. no. 3957.2]
500 mL ddH_2_O
Stir salt solution, filter through a 0.22‐µm filter (Millipore, cat. no. SEGPT0045), and autoclave.
Store at 4°C for sample preparation, or at room temperature.

### PBD (DNase I‐containing PBS/BSA)

125 µL DNase I aliquot (25 µg/mL final)
50 mL PBS/BSA [0.2% (w/v) BSA]

## COMMENTARY

### Critical Parameters

All buffers and sheath fluid of the flow cytometer should be autoclaved and/or at least sterile‐filtered through a 0.22‐µm filter. Keep samples on ice during sample preparation in Basic protocol 1. To avoid (cross) contamination during the entire workflow, use filter tips.

A centrifugation speed of 13,000 × g is crucial to not lose bacteria during washing steps. It is critical to use tubes that withstand high centrifugation forces and do not break. Vortex and spin down fluorochrome‐conjugated reagents to avoid transfer of aggregates and dye complexes to the staining mix during staining cocktail preparation.

Titrate all antibodies and lectins to determine the optimal reagent concentration and achieve the best signal‐to‐noise ratio. Use an excess of purified and unlabeled antibodies of the same isotype as the staining antibodies to avoid nonspecific binding of detection antibodies.

Perform blocking experiments with sugars to confirm specificity of lectin staining (see Suppl. Fig. 1). For biotinylated lectins, pre‐incubate with anti‐biotin antibody or perform sequential staining depending on the lectin. Evaluate whether sequential staining is appropriate. Include an external control (anchor sample) in Basic protocol 2 to control the staining procedure.

Perform regular instrument maintenance to ensure reliable performance and prevent biofilm formation in microfluidic lines. Perform washing cycles and bead controls in between to ensure stable sample acquisition.

### Troubleshooting Table

Common problems that may occur during the protocol, as well as their causes and potential solutions, are listed in Table 1.

**Table 1:** Troubleshooting Guide to Successfully Perform Microbiota Flow Cytometry

Problem	Possible Cause	Solution
30‐µm filter clogging	High amount of fiber and particulate matter in the sample or improper sample homogenization	Include an additional filter step through a 70‐µm filter prior to filtering through the 30‐µm filter.
No bacterial cell pellet after centrifugation step or the cell pellet detaches easily	Centrifugation speed or time insufficient	Spin down the cell suspension for 10 min at 13,000 × g and discard the supernatant quickly after centrifugation to prevent the cell pellet from dispersing.
Anchor sample showing unexpected staining pattern	Irregularities during staining process or changes in flow cytometer settings	Check flow cytometer settings and repeat Basic protocol 2.
Particles with high fluorescence intensity at the upper detection limit during sample acquisition	Dye aggregates	Vortex the fluorochrome‐conjugated reagents thoroughly and centrifuge them for 1 min at 17,000 × g to prevent dye aggregates from transferring into the staining solution.
High background of staining buffers	Undissolved salts or microbial contamination	Filter staining buffer through a 0.2‐µm filter prior to use.
High background of sheath fluid	Undissolved salts or biofilm formation in the microfluidic lines	Filter sheath fluid through a 0.2‐µm filter prior to use. Perform regular flow cytometer maintenance, including cleaning steps with antimicrobial agents (e.g., sodium hypochlorite) to prevent biofilm formation. Αpply in‐tank filters to filter the sheath fluid again. Optional: Apply a UV light decontamination unit to continuously irradiate the sheath fluid and prevent biofilm formation (Kirsch et al., 2022).
High bacterial event rate during acquisition	High density of bacterial cells within sample	Dilute the sample to reach an event rate of around 10,000 events/s.

### Statistical Analysis and Understanding Results

We described a comprehensive and modular workflow to successfully perform multi‐parametric microbiota flow cytometry starting from native stool, including a protocol to generate cryo‐preserved bacterial stocks and a surface staining procedure to profile bacteria coated with host‐endogenous immunoglobulins and sugar surface moieties. After sample acquisition and prior data analysis of multivariate data, we recommend checking for batch effects based on acquisition date, if data were acquired on multiple days. Recorded fcs files can be normalized according to the acquisition batch and corresponding anchor sample (biological control sample) using the CytoNorm R package (Quintelier et al., 2025). For bacterial cells, we apply the functions CytoNorm::*QuantileNorm.train(*nQ = 101, goal = “mean”*)* for model training and CytoNorm::*QuantileNorm.normalize()* for data normalization. An example of normalized data including anchor controls is shown in Supp. Figure 2.

For analysis of multi‐parametric microbiota flow cytometry, we suggest a workflow described in detail (Budzinski et al., 2025) to generate microbial fingerprints. Raw or normalized fcs files were read into R using the flowCore::read.flowSet() function (Hahne et al., 2009) and pre‐gated to exclude debris and electronical noise [events with MFI > 1 for side scatter (SSC) and forward scatter (FSC)] using the flowWorkspace package (see suppl. Fig. 3) (Finak et al., 2014). We recommend to train a self‐organized map (SOM) with 2025 data bins per panel and 3×10^5^ cells in total (subsampled from the respective samples applied to train the SOM) using the kohonen::som() function (Wehrens & Buydens, 2007). For phenotypic characterization, the trained SOM can be mapped to 3×10^4^ cells per sample after downsampling using the kohonen::map() function. Afterwards, each sample is described by the frequency of cells for each data bin/cluster. The microbiota phenotype (4050 clusters) comprises 2025 clusters derived from the immunoglobulin panel and lectin panel (bacterial cell surface sugar moieties), respectively. The microbiota phenotype can be visualized by β‐diversity projection (based on the Bray–Curtis dissimilarity matrix) using vegan::vegdist(method = ″bray) (Oksanen et al., 2024) to show phenotypic composition (Fig. 3A).

To refine the microbiota fingerprint into a microbiota signature, Wilcoxon rank sum test (Fig. 3, A2) can be applied to continue with clusters, which are statistically significant between two groups followed by recursive feature elimination (RFE, Fig. 3, A3) to systematically remove clusters with weak importance by iteratively training a predictive model and pruning the least contributing clusters until an optimal subset of clusters was achieved. For RFE, the caret::rfe() was used with caret:rfeControl(method = “cv”, number = 10) (Kuhn, 2008). The refined microbiota signature can be visualized in a β‐diversity projection (Fig. 3, A4), or the mean fluorescence intensity can be plotted for each stained marker (Fig. 3B). This projection also highlights the possibility of isolating clusters of interest by fluorescence‐activated cell sorting (FACS) for further investigation. The suggested data analysis workflow is also possible using the MicroBiotR package (Kang, 2025). Alternatively, other R packages and clustering algorithms can be applied to generate microbial fingerprints as described in earlier studies (Koch et al., 2013; Kupschus et al., 2023; Props et al., 2016; Rubbens et al., 2021b).

Additionally, our protocol outlined the quantification of the bacterial load. For data derived from the volumetric cell counting function, the following formula can be applied:

$$\begin{array}{ccc} \text{total cell count [cells]} & = & \frac{\text{cell count [cells]}}{\text{uptake volume}\;[\mu L]}\\ & & *\;\text{total volume}\;[\mu L] \end{array}$$

The cell count can be determined using analysis software such as FlowJo. For approaches including counting beads, a gating strategy is shown in Figure 4 to determine the cell count and number of counts using FlowJo software. The following formula can be used for calculation:

$$\begin{array}{ccc} & & \text{total cell count [cells]}\\ & & \;=\;\frac{\text{cell count [cells]}}{\text{counting beads count [beads]}}\\ & & \;*\;\text{counting beads concentration}\left[\frac{\text{beads}}{\mu L}\right]\\ & & \;*\;\text{total volume}\;[\mu L] \end{array}$$

The workflow described provides only phenotypic information and does not allow for conclusions regarding taxonomy. To address this issue, the populations of interest can be isolated by FACS and subjected to16S rRNA gene sequencing or shotgun metagenomics. However, the aerobic conditions during FACS may be harmful for strict anaerobic species, in case of post‐sorting cultivation. However, anaerobic sorting units have been described (Bellais et al., 2022).

### Time Considerations (Per Stool Sample)

Preparation of cryo‐preserved stocks from native stool requires ∼60 min. Please note that time does not increase linearly with the number of samples. Sample staining takes ∼2–2.5 h. Instrument setup and alignment need 60 min, depending on the device. Acquisition time per set of tests per donor is ∼10 –15 min (including unstained test, DNA‐only test, immunoglobulin‐stained sample, lectin‐stained sample, and cell counting).

### Author Contributions

**Toni Sempert**: Methodology; investigation; writing original draft; writing review and editing. **Lisa Budzinski**: Methodology; investigation; writing review and editing. **Robin Kempkens**: Methodology; investigation; writing review and editing. **Gi‐Ung Kang**: Methodology; investigation. **René Maier**: methodology; investigation. **Hyun‐Dong Chang**: Resources; funding acquisition; supervision; project administration; writing review and editing.

### Conflict of Interest

The authors declare no conflict of interest.

## Supporting information




*Supplementary Figure 1. Blocking assay to validate lectin target sugar specificity. Exemplary data to test the binding specificity of Peanut Agglutinin (PNA) for galactose, mannose and N‐acetylglucosamine in concentrations from 1 mM to 100 mM. Histograms visualize geometric mean fluorescence intensity of PNA+ cells of PNA single stain (unblocked test, positive control), DNA‐dye‐only test (background) as well tests blocked with varying target sugar concentration to confirm binding specificity*.

*Supplementary Figure 2. Batch‐effect correction using anchor samples with CytoNorm V2. PCoA projection based on Bray–Curtis dissimilarity matrix for healthy individuals (filled circle) and external anchor samples (triangles) of two cohorts measured 6–12 months apart before (A) and after (B) batch‐effect correction. Data were corrected based on acquisition batch. R2 and PERMANOVA test for the comparison of batch 1 (light green) vs batch 2 (purple)*.

*Supplementary Figure 3. Representative gating strategy for microbiota phenotyping analysis. The unstained stained sample is first used to establish a cell gate on SSC (y‐axis) vs. FSC (x‐axis) to exclude electronic noise and cellular debris. An analysis gate is then defined on FSC (x‐axis) vs. Hoechst33342 fluorescence (y‐axis) to gate on bacterial cells of interest using an overlay of unstained sample (grey) and DNA‐only stained sample (orange). The identical gating hierarchy is applied to samples stained with the anti‐human immunoglobulin/lectin cocktails. 2D overlays (DNA‐only stained sample, orange; panel‐stained sample, black) illustrate marker‐specific staining on FSC (x‐axis) vs. marker of interest (y‐axis) for each antibody or lectin*.

## Data Availability

Data presented in the figures are available upon request.
